# The Ecologic Validity of Fructose Feeding Trials: Supraphysiological Feeding of Fructose in Human Trials Requires Careful Consideration When Drawing Conclusions on Cardiometabolic Risk

**DOI:** 10.3389/fnut.2015.00012

**Published:** 2015-05-06

**Authors:** Vivian L. Choo, John L. Sievenpiper

**Affiliations:** ^1^Department of Nutritional Sciences, Faculty of Medicine, University of Toronto, Toronto, ON, Canada; ^2^Toronto 3D Knowledge Synthesis and Clinical Trials Unit, Clinical Nutrition and Risk Factor Modification Centre, St. Michael’s Hospital, Toronto, ON, Canada; ^3^Division of Endocrinology and Metabolism, Department of Medicine, St. Michael’s Hospital, Toronto, ON, Canada

**Keywords:** fructose, HFCS, dose, cardiometabolic risk, meta-analysis

## Abstract

**Background:**

Select trials of fructose overfeeding have been used to implicate fructose as a driver of cardiometabolic risk.

**Objective:**

We examined temporal trends of fructose dose in human controlled feeding trials of fructose and cardiometabolic risk.

**Methods:**

We combined studies from eight meta-analyses on fructose and cardiometabolic risk to assess the average fructose dose used in these trials. Two types of trials were identified: (1) substitution trials, in which energy from fructose was exchanged with equal energy from other carbohydrates and (2) addition trials, in which energy from fructose supplemented a diet compared to the diet alone.

**Results:**

We included 64 substitution trials and 16 addition trials. The weighted average fructose dose in substitution trials was 101.7 g/day (95% CI: 98.4–105.1 g/day), and the weighted average fructose dose in addition trials was 187.3 g/day (95% CI: 181.4–192.9 g/day).

**Conclusion:**

Average fructose dose in substitution and addition trials greatly exceed national levels of reported fructose intake (49 ± 1.0 g/day) (NHANES 1977–2004). Future trials using fructose doses at real world levels are needed.

## Introduction

With the increase in high-fructose corn syrup (HFCS) consumption since 1970s, there has been rising interest in the role of sugars toward the development of cardiometabolic diseases (1). Particular attention has focused on the “fructose hypothesis,” which suggests that the metabolic and endocrine responses unique to fructose are the main drivers in the etiology of obesity, diabetes, and cardiometabolic risk (2, 3). While this perspective is well supported by lower quality evidence from ecological studies (4) and animal models (5–7), it is not well supported by the highest level of evidence from controlled trials in humans (8–13).

A main limitation of these trials has been the use of extreme levels of fructose feeding not representative of real world conditions. The present analysis aims to quantify the dose of fructose used in trials assessing the effects of fructose and cardiometabolic risk, and compare it to national levels of fructose consumption in the United States at the average and 95th percentile levels of intake based on the National Health and Nutrition Examination Survey (NHANES 1977–2004) (14).

## Materials and Methods

We collated studies previously identified in a series of meta-analyses and systematic reviews of the effects of fructose on various cardiometabbolic endpoints (8–13). We included controlled dietary trials across all populations investigating the effect of fructose on fasting blood lipids (Chiavaroli et al., unpublished study), postprandial triglycerides (13), blood pressure (9), glycemic control (Cozma et al., unpublished study), uric acid (11), non-alcoholic fatty liver disease (NAFLD) (12), body weight using mixed forms of fructose (solid, liquid, mixed) (10), and body weight from fructose-containing sugars-sweetened beverages only (Choo et al., unpublished study). Trials lasting <7 days, using intravenous administration or possessing unsuitable endpoints or comparators were excluded. Two types of trials were identified for the purposes of this analysis-substitution trials, in which fructose was exchanged for equal amounts of energy from other carbohydrates, or addition trials, in which a control diet was supplemented with additional energy from fructose compared to the control diet alone without the excess energy. Duplicate studies between meta-analyses were removed, and fructose dose data were extracted from each study when available and reported in grams per day. A weighted average fructose dose used across all studies was calculated according to the sample size of each trial, and reported as a mean and 95% confidence interval.

## Results

**Figure 1:**
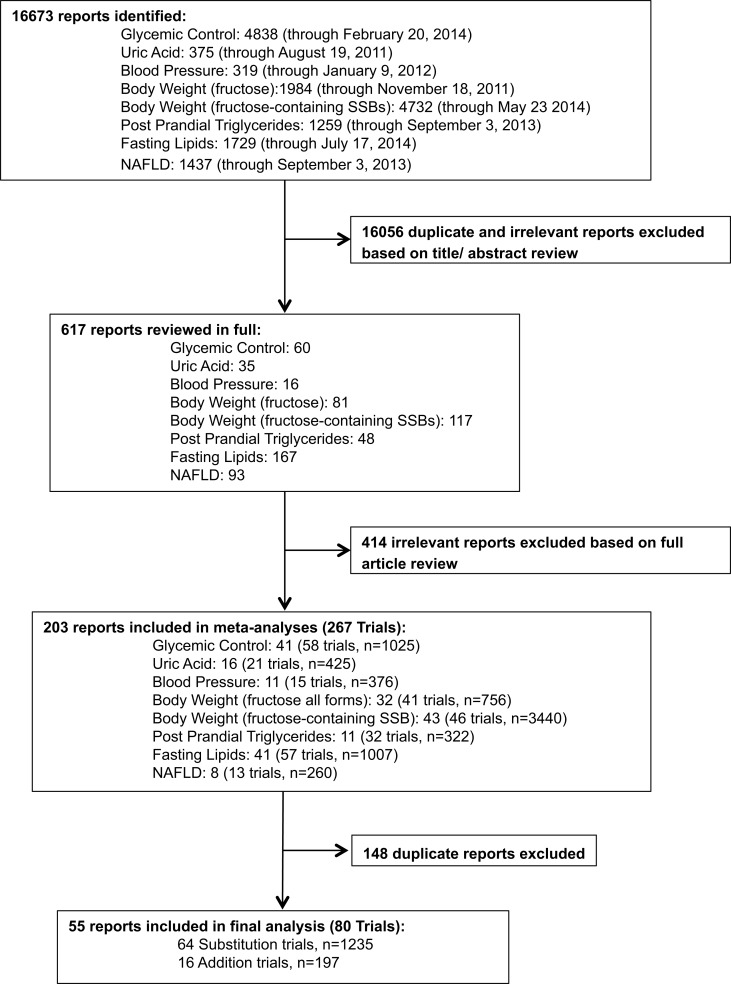
**Systematic search and selection strategy**. Flow of literature for eight separate searches of the effect of fructose on: glycemic control (fasting blood glucose, fasting blood insulin, HbA1c), uric acid, blood pressure, body weight (fructose), body weight (fructose-containing sugars-sweetened beverages, post prandial triglycerides, fasting lipids, and NAFLD.

The search and selection process can be found in Figure 1. A total of 16,673 reports were identified between all meta-analyses, and 203 reports (267 trials) were included after excluding reports based on title and abstract. After combining eligible trials and removal of duplicates from the meta-analyses, 64 substitution trials (1235 participants) and 16 addition trials (197 participants) were included in this analysis.

### Trial characteristics

Table 1 provides a detailed summary of trial characteristics. There were 64 substitution trials involving 1235 participants (15–63) and 16 addition trials involving 197 participants (23, 30, 49, 50, 53, 54, 56, 58, 59, 64–66). Sample sizes of substitution and addition trials tended to be small [median number of participants, 12.5 (IQR: 9–24) and 12.5 (IQR: 8–16) for substitution and addition trials, respectively]. A majority of trials used a crossover design (69 and 94% of substitution and addition trials, respectively). Participants in substitution trials tended to be middle aged males and females [55% males; median age, 39.5 years (IQR: 23.4–53 years)], whereas participants in addition trials tended to be younger males [81% males; median age, 24.7 years (IQR: 23.5–33.9 years)]. Study duration was relatively short in both types of trials [median, 4 weeks (IQR: 2–6 weeks) and median 1.5 weeks, (IQR: 1–4 weeks) in substitution and addition trials, respectively] and predominantly took place in the United States for substitution trials and Europe for addition trials under an outpatient setting. Comparators in substitution trials included starch (30%), glucose (26%), sucrose (8%), d-maltose (3%), galactose (2%), and HFCS (1%) and comparators in all addition trials were diet alone.

**Table 1:** **Characteristics of trials investigating the effect of fructose on cardiometabolic risk**.

Reference	Subjects^a^	Age (years)	Setting	Design	Feeding control^b^	Randomization	Fructose dose^c^	Fructose form^d^	Comparator	Diet^e^	Follow-up	MQS^f^	Energy Balance	Funding sources^g^
**SUBSTITUTION TRIALS**														
(15)	5 HTG (3M:2F) 4 N (3M:1F)	42.8 ± 14.2	IP/OP, Israel	C	Met	No	300 g/d (55% E)	Mixed	Starch	77:05:18	~24 d	7	Neutral	Agency

(16) (Study 1)	3 HTG	19 ± 0	IP, Australia	C	Met	No	~255 g/d (50–52% E)	Mixed	Glucose	77:09:14	1 wk	6	Neutral	Agency

(16) (Study 2)	2 HTG	19 ± 0	IP, Australia	C	Met	No	~255 g/d (52–55% E)	Mixed	Glucose	77:09:14	1 wk	6	Neutral	Agency

(17)	16 DM1	10 (2–16)	OP, Finland	C	Supp	No	~40 g/d (20% E)	Mixed	Starch	45:35:20	1 wk	4	Neutral	Industry

(18)	10 type 4 HTG (5 DM2)	53.5 (26-67)	IP, Finland	C	Met	Yes	~77.5 g/d (~17% E)	Liquid	Starch, sucrose	45:35:20	10–20 d	6	Neutral	Agency

(19)	10 DM1 (5M:5F)	25.5 (19–70)	IP, Finland	C	Met	No	75 g/d (15% E)	Mixed	Starch	40:40:20	10 d	7	Neutral	Agency

(20)	12 N (8M:4F)	(20–26)	IP, Germany	C	Met	No	162 g/d (~33% E)	Liquid	Glucose, sucrose	90:00:10	10 d	7	Neutral	–

(21)	68 N	(13–55)	OP, Finland	P	Dietary Advice	No	70 g/d (~14% E)	Mixed	Sucrose	-	72 wks	5	Neutral	–

(22) (LC)	4 HTG (4M:0F)	48 ± 8.8	IP, USA	C	Met	No	~39.5 g/d (9% E)	Liquid	d-Maltose	45:40:15	2 wks	7	Neutral	Agency and industry

(22) (HC)	4 HTG (4M:0F)	48 ± 8.8	IP, USA	C	Met	No	~122 g/d (17%E)	Liquid	d-Maltose	85:00:15	2 wks	4	Neutral	Agency and industry

(22)	2 DM2 (2M:0F)	41 ± 1.4	IP, USA	C	Met	No	~40 g/d (9% E)	Liquid	d-Maltose	45:40:15	2 wks	7	Neutral	Agency and industry

(23)	15 N	(21–35)	OP, Denmark	P	Supp	Yes	+250 g/d (+50% E)	Liquid	Glucose	44:38:18	1 wk	6	Positive	Agency and industry

(24)	16 type 4 HTG	57 (38–80)	OP, Poland	C	Supp	No	80 g/d	Liquid	Starch	45:50:15	28 d	7	Neutral	–

(25) – N (HF)	12 N (12M:0F)	39.8 ± 8.3	IP/OP, USA	C	Met	No	101.3 g/d (15% E)	Solid	Starch	43:42:15	5 wks	8	Neutral	–

(25) – N (LF)	12 N (12M:0F)	39.8 ± 8.3	IP/OP, USA	C	Met	No	50.6 g/d (7.5% E)	Solid	Starch	43:42:15	5 wks	8	Neutral	–

(25) – HI (HF)	12 HI (12M:0F)	39.5 ± 7.3	IP/OP, USA	C	Met	No	101.3 g/d (15% E)	Solid	Starch	43:42:15	5 wks	8	Neutral	–

(25) – HI (LF)	12 HI (12M:0F)	39.5 ± 7.3	IP/OP, USA	C	Met	No	50.6 g/d (7.5% E)	Solid	Starch	43:42:15	5 wks	8	Neutral	–

(26)	8 N (4M:4F)	26.7 (20–32)	IP/OP, USA	C	Met	Yes	~79 g/d (14% E)	Liquid	Sucrose	~43:40:17	2 wks	8	Neutral	Agency

(27)	11 N (4M:7F)	39.5 ± 11.4	IP/OP, USA	C	Met	No	~81 g/d (13.2% E)	Mixed	Sucrose	55:30:15	2 wks	7	Neutral	Agency and industry

(28)	12 DM1 (6M:6F) 12 DM2 (5M:7F)	23 (15–32) 62 (36–80)	OP, USA	C	Met	Yes	~137 g/d (21% E)	Mixed	Starch	55:30:15	8 d	8	Neutral	Industry

(29)	7 DM2 (3M:4F)	50.9 ± 8.4	IP/OP, USA	C	Met	No	~98 g/d (13.2% E)	Mixed	Sucrose	55:30:15	2 wks	7	Neutral	Agency and industry

(30) EXP 1	23 OW/OB	22.2	OP, France	P	Met	Yes	36 g/d (25%E)	Liquid	Glucose, galactose	25:50:25	2 wks	8	Negative	Industry

(30) EXP 2	18 OW/OB	22.2	OP, France	P	Met	Yes	36 g/d (25%E)	Liquid	Glucose, galactose	25:50:25	2 wks	8	Negative	Industry

(31)	10 DM2	64.4 (54–71)	OP, Ireland	C	Supp	No	55 g/d (11.6% E)	Liquid	Starch	42:38:20	4 wks	7	Neutral	Industry

(32)	18 DM2 (3M:15F)	57 ± 3.0	OP, USA	P	Supp	Yes	60 g/d (10% E)	Mixed	Starch	50:35:15	12 wks	8	Neutral	Agency and industry

(33)	8 DM2 (5M:3F)	40 ± 6.9	OP, France	C	Supp	Yes	30 g/d (8% E)	Mixed	Starch	50:30:20	8 wks	8	Neutral	Agency and industry

(34) – NGT	9 N (3M:6F)	48	OP, USA	C	Supp	No	~79 g/d (15% E)	Mixed	Glucose	~53:32:16	4 wks	8	Neutral	–

(34) – IGT	9 IGT (3M:6F)	53	OP, USA	C	Supp	No	~64 g/d (15% E)	Mixed	Glucose	~53:32:16	4 wks	8	Neutral	–

(35)	14 DM2 (14M:0F)	60 ± 4 (54–71)	IP/OP, USA	C	Met/Supp	No	~55 g/d (12% E)	Mixed	Starch	53:27:20	23 wks	8	Neutral	Agency and industry

(36)	13 DM2 (5M:8F)	54 ± 11	OP, USA	C	Supp	Yes	60 g/d (7.5% E)	Mixed	Starch	50:35:15	26 wks	8	Neutral	Agency and industry

(37)	10 IR (10M: 0F)	47	IP, USA	C	Met	No	167 g/d (20% E)	Solid	Starch	51:36:13	5 wks	4	Neutral	–

(38)	8 DM2 (4M:4F)	55 ± 11.2	IP, USA	P	Met	No	~100 g/d (13% E)	Mixed	Sucrose	55:30:15	12 wks	6	Neutral	Agency and industry

(67)	14 DM1, 6 DM2	46.9 ± 13.1	OP, France	P	Supp	Yes	~25 g/d (5% E)	Mixed	Starch, sucrose	55:30:15	52 wks	7	Neutral	Agency and industry

(68)	6 DM2 (4M:2F)	53.7 ± 10.2	IP, USA	C	Met	No	~100 g/d (13% E)	Mixed	Sucrose	55:30:15	100 d	4	Neutral	Agency and industry

(39)	6 DM1 (3M:3F) 12 DM2 (4M:8F)	23 (18–34) 62 (40–72)	OP, USA	C	Met	Yes	~120 g/d (20% E)	Mixed	Starch	55:30:15	4 wks	8	Neutral	Agency

(40)	14 N (7M:7F)	34 (19–60)	IP/OP, USA	C	Met	Yes	~120 g/d (20% E)	Mixed	Starch	55:30:15	4 wks	8	Neutral	Agency

(41)	10 DM2 (4M:6F)	61 ± 9.5	IP, Finland	C	Met	Yes	~55 g/d (10% E)	Liquid	Starch	50:30:20	4 wks	9	Neutral	Agency

(42)	16 DM2 (7M:9F)	54.2 ± 9.2	OP, Brazil	C	Supp	No	63.2 g/d (20% E)	Liquid	Starch, sucrose	55:30:15	4 wks	7	Neutral	Industry

(43)	24 N (12M:12F)	41.3 ± 20.0	OP, USA	C	Met	Yes	85 g/d (17% E)	Mixed	Glucose	55:30:15	6 wks	9	Neutral	Agency

(44) – P1	24 N (12M:12F)	14.6 ± 1.2	OP, USA	P	Met	Yes	64.19 g/d (12% E)	Mixed	Starch	30:55:15	1 wk	9	Neutral	Agency and industry

(44) – P2	12 N (6M:6F)	14.8 ± 1.32	OP, USA	C	Met	Yes	~151.32 g/d (24% E)	Mixed	Starch	60:25:15	1 wk	9	Neutral	Agency and industry

(45)	12 N (6M:6F)	15.3 ± 0.8	OP, USA	C	Met	Yes	128.5g/d (40% E)	Mixed	Starch	60:25:15	8 d	9	Neutral	Agency and industry

(46)	25 DM2	62.3 ± 10.1	OP, Israel	P	Supp	Yes	22.5 g/d (4.5% E)	Liquid	Starch	–	12 wks	5	Neutral	–

(47)	6 OB (3M:3F)	15.2 ± 1.22	OP, USA	C	Met	Yes	~149.1 g/d (24% E)	Mixed	Starch	60:25:15	1 wk	9	Neutral	Agency and industry

(48)	7 OW/OB (0M:7F)	(50–72)	IP, USA	C	Met	No	~125 g/d (25% E)	Liquid	Starch	55:30:15	10 wks	7	Neutral	Agency

(49)	32 OW/OB (16M:16F)	53	IP/OP, USA	P	Met/Supp	No	~182 g/d (+ 25% E)	Liquid	Glucose	55:30:15	10 wks	6	Positive	Agency

(50)	11 N (11M:0F)	24.6 ± 2.0	OP, Switzerland	C	Met	Yes	~+213 g/d (+ 35% E)	Liquid	Glucose	55:30:15	1 wk	8	Positive	Agency

(51) (LF)	29 N (29M:0F)	26.3 ± 6.6	OP, Switzerland	C	Supp	Yes	~40 g/d (7% E)	Liquid	Glucose, starch	51:14:35	3 wks	9	Positive	Agency

(51) (HF)	29 N (29M:0F)	26.3 ± 6.6	OP, Switzerland	C	Supp	Yes	~80 g/d (13% E)	Liquid	Glucose, sucrose	55:13:32	3 wks	9	Positive	Agency

(52)	131 OW/OB (29M:102F)	38.8 ± 8.8	OP, Mexico	P	Dietary advice	Yes	~60 g/d (13% E)	Solid	Starch	55:30:15	6 wks	9	Negative	Agency

(53)	20 N (12M:8F)	30.5 ± 8.93	OP, Germany	P	Supp	Yes	~+150 g/d (+ 22% E)	Liquid	Glucose	50:35:15	4 wks	7	Positive	Agency

(54)	32 OW/OB (16M:16F)	54 ± 8	IP/OP, USA	P	Met/Supp	No	~+182 g/d (+ 25% E)	Liquid	Glucose	55:30:15	10 wks	6	Positive	Agency

(54)	48 N (27M:21F)	27.6 ± 7.1	IP/OP, USA	P	Met/Supp	No	~+168 g/d (+ 25% E)	Liquid	Glucose HFCS	55:30:15	2 wks	6	Positive	Agency

(55)	28 CKD (17M:11F)	59 ± 15	OP, Poland	C	Dietary advice	No	~56 g/d (10% E)	Mixed	Starch	55:30:15	6 wks	8	Neutral	Agency

(56)	31 OW/OB (16M:15F)	53.7 ± 8.1	IP/OP, USA	P	Met/Supp	No	~+182 g/d (+25% E)	Liquid	Glucose	55:30:15	10 wks	6	Positive	Agency

(57)	9 N (9M:0F)	22.7 ± 1.8	OP, Switzerland	C	Supp	Yes	~80 g/d (+13% E)	Liquid	Glucose sucrose	55:31:14	3 wks	9	Positive	Agency

(58) – (NEB)	32 OW/OB (32M:0F)	33.9 ± 10.0	OP, UK	P	Met/Supp	Yes	~204 g/d (25% E)	Liquid	Glucose	55:30:15	8 wks	10	Neutral	Agency

(58) – (PEB)	32 OW/OB (32M:0F)	33.9 ± 10.0	OP, UK	P	Met/Supp	Yes	~+204 g/d (+25% E)	Liquid	Glucose	55:30:15	8 wks	10	Positive	Agency

(59)	28 N (28M:0F)	22.5 ± 1.6	OP, Switzerland	P	Supp	Yes	~212 g/d (+24% E)	Liquid	Glucose	–	7 d	9	Positive	Agency

(60)	9 N (4M:5F)	20.9 ± 2	OP, USA	C	Met	Yes	~129 g/d (25% E)	Liquid	Glucose	50:34:16	8 d	8		–

(61)	40 N (40M:20F)	17.9 ± 1.9	OP, USA	C	Supp	Yes	~50 g/d (+10% E)	Liquid	Glucose	–	2 wks	7	Positive	Agency

(62)	21 OW (11M:10F)	13.5 ± 2.5	OP, USA	P	Supp	Yes	~+99 g/d (+19.8% E)	Liquid	Glucose	–	4 wks	5	Neutral	Agency

(63)	73 OW (0M:73F)	39.7 ± 8.6	OP, Denmark	P	Supp	Yes	~+60 g/d (+13.6% E)	Liquid	Glucose	45:34:21	4 wks	9	Positive	Agency

(61)	7 OW (3M:4F)	18 ± 0.4	OP, USA	C	Supp	Yes	~+50 g/d (+6.7% E)	Liquid	Glucose	–	2 wks	8	Positive	Agency
**ADDITION TRIALS**														
(23)	8 N	21–35	OP, Denmark	C	Supp	No	~250 g/d (~+50% E)	Liquid	Diet alone	44:38:18	1 wk	5	Positive	Agency and industry

(30) EXP 2	14 OW/OB	22.2	OP, France	P	Met	Yes	~+100 g/d (+97% E)	Liquid	Diet alone	0:35:65	2 wks	8	Negative	Industry

(64)	7 N (7M:0F)	24.70 ± 3.44	OP, Switzerland	C	Supp	No	~+104 g/d (+18% E)	Liquid	Diet alone	55:30:15	4 wks	7	Positive	Agency

(65) (N)	8 N (8M:0F)	24.5 ± 4.5	OP, Switzerland	C	Supp	Yes	~213 g/d (+35% E)	Liquid	Diet alone	55:30:15	7 d	9	Positive	Agency and industry

(65)	16 OFFDM2 (16M:0F)	24.7 ± 5.2	OP, Switzerland	C	Supp	Yes	~220 g/d (+35% E)	Liquid	Diet alone	55:30:15	1 wk	8	Positive	Agency and industry

(49)	17 OW/OB (9M:8F)	52.5 ± 9.2	IP/OP, USA	C	Met/Supp	No	~182 g/d (25% E)	Liquid	Diet alone	55:30:15	10 wks	6	Positive	Agency

(50)	11 N (11M:0F)	24.6	OP, Switzerland	C	Met/Supp	Yes	~213 g/d (+35%E)	Liquid	Diet alone	55:30:15	7 d	8	Positive	Agency

(66)	8 N (8M:0F)	24.8 ± 3.2	OP, Switzerland	C	Supp	No	~+212 g/d (+35% E)	Liquid	Diet alone	55:30:15	1 wk	6	Positive	Agency

(53)	10 N (7M:3F)	32.8 ± 9.3	OP, Germany	C	Supp	No	~+150 g/d (+22% E)	Liquid	Diet alone	50:35:15	4 wks	6	Positive	Agency

(54)	17 OW/OB (9M:8F)	52.5 ± 9.3	IP/OP, USA	C	Met/Supp	No	~+182 g/d (+25% E)	Liquid	Diet alone	55:30:15	10 wks	5	Positive	Agency

(54)	16 N (9M:7F)	28.0 ± 6.8	IP/OP, USA	C	Met/Supp	No	~+168 g/d (+25% E)	Liquid	Diet alone	55:30:15	2 wks	6	Positive	Agency

(56)	16 OW/OB (9M:7F)	52.5 ± 9.3	IP/OP, USA	C	Met/Supp	No	~+182 g/d (+25% E)	Liquid	Diet alone	55:30:15	10 wks	5	Positive	Agency

(58)	15 OW/OB (15M:0F)	35.0 ± 11.0	OP, UK	C	Supp	No	~+203 g/d (+25% E)	Liquid	Diet alone	55:30:15	2 wks	8	Positive	Agency

(59) (F1.5)	7 N (7M:0F)	22.5 ± 1.6	OP, Switzerland	C	Supp	Yes	~+104 g/d (~+14% E)	Liquid	Diet alone	–	7 d	9	Positive	Agency

(59) (F3.0)	17 N (17M:0F)	22.5 ± 1.6	OP, Switzerland	C	Supp	Yes	~212 g/d (+ ~24%E)	Liquid	Diet alone	–	7 d	10	Positive	Agency

(59) (F4.0)	10 N (10M:0F)	22.5 ± 1.6	OP, Switzerland	C	Supp	Yes	~293 g/d (~30% E)	Liquid	Diet alone	–	7 d	11	Positive	Agency

*C, crossover; CKD, chronic kidney disease; d, days; DM1, person with diabetes mellitus type-1; DM2, person with diabetes mellitus type-2; E, energy; F, female EXP, experiment; F1.5/3.0/4/0, fructose at 1.5, 3.0, or 4.0 kg/day; HC, high carbohydrate; HF, high fructose; HI, hyperinsulinemic; HTG, hypertriglyceridemic; IGT, impaired glucose tolerance; IP, inpatient; IR, insulin resistant; LC, low carbohydrate; LF, low fructose; M, male; N, normal; NEB, neutral energy balance; OFFDM2, offspring of persons with type-2 diabetes mellitus; OP, outpatient; OW/OB, overweight/obese; P, parallel; PEB, positive energy balance; P1/2, protocol 1/2; Supp, supplement; UK, United Kingdom; USA, United States of America; wks, weeks*.

*^a^We applied an intention-to-treat analysis to Thorburn et al. carrying forward the baseline data of a participant that dropped out of the study halfway*.

*^b^Metabolic (Met) feeding control represents the provision of all meals, snacks, and study supplements (test sugars and foods) consumed during the study under controlled conditions. Supplement (Supp) feeding control represents the provision of study supplements. Certain studies provided partial-metabolic (Met/Supp) feeding, containing a metabolic and a supplemental period. Dietary advice represents the provision of counseling on the appropriate test and control diets*.

*^c^Doses were administered on a g/day, % energy, or g/kg body weight basis. Doses preceded by “~” represent an average dose calculated based on the average reported energy intake or weight of participants. If these data were not available, then the average dose was based on a 2000-kcal intake or 70-kg weight. Plus signs indicated excess energy provided by fructose*.

*^d^Fructose was provided in one of three forms: (1) liquid, where all or most of the fructose was provided as beverages or crystalline fructose to be added to beverages; (2) solid form, where fructose was provided as solid foods; or (3) mixed, where all or most of the fructose was provided as a combination of beverages, solid foods, and/or crystalline fructose*.

*^e^Values for energy are in the form of carbohydrate: fat: protein. “−” indicates that the information was not available*.

*^f^Trials with a score ≥8 were considered to be of higher quality, according to the Heyland Methodological Quality Score*.

*^g^Agency funding includes those from government, university, or non-profit health agency sources*.

### Fructose dose

**Figure 2:**
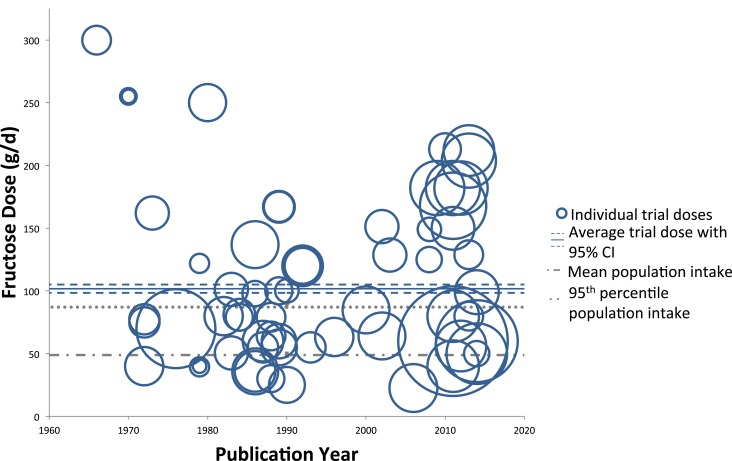
**Trends of fructose dose in substitution trials**. Individual trials are plotted based on date of publication and fructose dose used. Sample size of each trial is represented by the size of its respective circle. The weighted average fructose dose across all substitution trials was 101.7 g/day (95% CI: 98.4–105.1 g/day), indicated by the solid and dashed blue lines.

**Figure 3:**
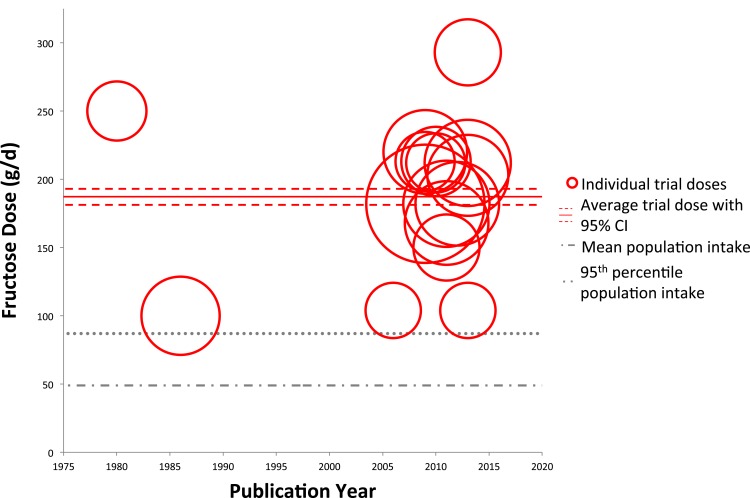
**Trends of fructose dose in addition trials**. Individual trials are plotted based on date of publication and fructose dose used. Sample size of each trial is represented by the size of its respective circle. The weighted average fructose dose across all addition trials was 187.3 g/day (95% CI: 181.4–192.9 g/day), indicated by the solid and dashed red lines.

Figures 2 and 3 show trends of fructose dose in substitution and addition trials plotted against the average and 95th percentile intakes of fructose in the United States (49 ± 1.0 and 87 ± 4.0 g/day, respectively). Substitution trials were conducted from 1966 to 2014 with most conducted during 1980s and a recent resurgence in 2010s, while the addition trials were conducted from 1980 to 2013 with most conducted after the mid 2000s. The weighted average fructose dose in substitution trials was two times higher than reported average population intake levels [101.7 g/day (95% CI: 98.4–105.1 g/day)], whereas the weighted average fructose dose in the addition trials was much greater, at ~3.7 times the amount of the reported average population intake levels [187.3 g/day (95% CI: 181.4–192.9 g/day)].

## Discussion

This analysis, which combined the trials identified from eight meta-analyses, aimed to examine the trends of fructose dose in controlled dietary trials assessing the effects of fructose on various cardiometabolic outcomes. We identified 64 substitution trials, in which fructose was provided in isocaloric substitution for other carbohydrate sources (usually starch), and 16 addition trials, in which fructose supplemented diets with excess energy compared to the same diets without the excess energy. The average weighted fructose dose was 101.7 g/day (95% CI: 98.4–105.1 g/day) in substitution trials from 1966 to 2014, whereas the average weighted fructose dose was nearly twice as high at 187.3 g/day (95% CI: 181.4–192.9 g/day) in the 16 addition trials from 1980 to 2013.

There were differences observed in the temporal trends between substitution and addition trials. Most substitution trials were conducted in 1980s with a resurgence that followed in 2010s. The reason for this pattern is unclear. A growing interest in fructose trials early on may have reflected the initial interest in fructose as a potentially beneficial alternative sweetener (69–71). By controlling for energy, substitution trials provided a rigorous study design, which allowed for the assessment of whether fructose had a unique set of metabolic or endocrine responses beyond its energy across a wide dose range. The emergence of the addition trials in 2000s may have grown out of the consistent lack of effect or even the benefit (glycemic control) seen in the substitution trials (8) and the concern stimulated by the ecological analysis of Bray et al. (4) linking fructose from HFCS with the epidemic of overweight and obesity. The recent resurgence of substitution trials in 2010s appears to have been to reconcile the role of energy from that of fructose in the addition trials. To test whether overfeeding of fructose differs from overfeeding of any other macronutrient (usually glucose or starch), these trials have compared fructose with other sources of carbohydrate under conditions of matched overfeeding.

Irrespective of any control for energy, the levels of intake observed in the available trials has been well beyond population levels of consumption. Compared to levels of reported fructose intake assessed by the National Health and Examination Survey in the United States (NHANES 1977–2004), the doses used in both the substitution and the addition trials exceeded the average and 95th percentile levels of fructose consumption (49 ± 1.0 and 87 ± 4.0 g/day, respectively). Furthermore, all addition trials used doses of fructose above the 95th percentile of reported intake, with the weighted average dose more than double that amount. While the present analysis suggests that these trials using supraphysiological doses of fructose feeding are not representative of levels normally consumed in the diet, the important caveat remains that underreporting from national population intake surveys, such as NHANES, may underestimate the actual amount of fructose consumed (72). However, taking into consideration the potential for underreporting when interpreting calculated trial means compared to reported population means, if an estimated level of 50% underreporting were present (average and 95th percentile fructose intake of 100 and 172 g/day, respectively), the fructose dose in substitution trials would reach levels representative of true dietary intake [101.7 g/day (95% CI: 98.4–105.1 g/day)], while supraphysiological doses of fructose in addition trials would still persist [187.3 g/day (95% CI: 181.4–192.9 g/day)]. Another important consideration is that fructose consumption has been changing with time in NHANES. HFCS (a main proxy for fructose consumption) availability has been declining since it peaked in 1999 (73). Variability of fructose consumption over time should be taken into consideration when predicting the true population average intake.

The implications of our findings suggest a potential lack of ecological validity when drawing conclusions from addition trials using unrealistically high doses of fructose. As with the excess consumption of any macronutrient, an adverse effect on cardiometabolic risk factors may be irrelevant under levels of normal dietary consumption and lead to unnecessary concern and confusion regarding the safety of fructose. Two trial designs have helped to clarify whether adverse effects relate to excess energy (either from fructose or any macronutrient in general) or specific metabolic and endocrine properties inherent to fructose itself. In a series of systematic reviews and meta-analyses of controlled trials to determine the effect of fructose on various cardiometabolic outcomes, a consistent signal for harm has only been shown in the addition trials (8–10, 12, 13). Substitution trials have failed to show differences in body weight (10), fasting triglycerides (74), postprandial triglycerides (13), uric acid (9), glucose, insulin (8), or markers of NAFLD (12) with improvements seen in blood pressure (9) and glycemic control (8, 75). These findings hold even under conditions of overfeeding as long as the excess energy is matched. The one exception may be for an effect on fasting triglycerides at a high dose threshold as seen in some subgroup analyses (76, 77). Taken together, these findings suggest that fructose appears to be a determinant of cardiometabolic risk only in as much as it contributes to excess energy in the diet.

## Conclusion

Most trials on fructose and cardiometabolic risk have used doses of fructose well beyond reported population levels of intake. While such high doses may be useful for determining a cause-effect relationship, replication of these studies using fructose doses closer to dietary levels are warranted and could help to establish a threshold beyond which excess energy from fructose demonstrate a signal for harm under real world conditions.

## Conflict of Interest Statement

VC has received research support from the Canadian Institutes of Health Research (CIHR). She also received a summer student scholarship from the Canadian Sugar Institute. JS has received research support from the Canadian Institutes of health Research (CIHR), Calorie Control Council, American Society of Nutrition (ASN), The Coca-Cola Company (investigator initiated, unrestricted), Dr. Pepper Snapple Group (investigator initiated, unrestricted), Pulse Canada, and The International Tree Nut Council Nutrition Research and Education Foundation. He has received reimbursement of travel expense, speaker fees, and/or honoraria from the American Heart Association (AHA), American College of Physicians (ACP), American Society for Nutrition (ASN), National Institute of Diabetes and Digestive and Kidney Diseases (NIDDK), Canadian Diabetes Association (CDA), Canadian Nutrition Society (CNS), University of South Carolina, University of Alabama at Birmingham, Oldways Preservation Trust, Nutrition Foundation of Italy (NFI), Calorie Control Council, Diabetes and Nutrition Study Group (DNSG) of the European Association for the Study of Diabetes (EASD), International Life Sciences Institute (ILSI) North America, International Life Sciences Institute (ILSI) Brazil, Abbott Laboratories, Pulse Canada, Canadian Sugar Institute, Dr. Pepper Snapple Group, The Coca-Cola Company, Corn Refiners Association, World Sugar Research Organization, Dairy Farmers of Canada, Società Italiana di Nutrizione Umana (SINU), and C3 Collaborating for Health. He has *ad hoc* consulting arrangements with Winston & Strawn LLP, Perkins Coie LLP, and Tate & Lyle. He is on the Clinical Practice Guidelines Expert Committee for Nutrition Therapy of both the Canadian Diabetes Association (CDA) and European Association for the study of Diabetes (EASD), as well as being on an American Society for Nutrition (ASN) writing panel for a scientific statement on sugars. He is a member of the International Carbohydrate Quality Consortium (ICQC) and Board Member of the Diabetes and Nutrition Study Group (DNSG) of the EASD. He serves an unpaid scientific advisor for the International Life Science Institute (ILSI) North America, Food, Nutrition, and Safety Program (FNSP) and the Committee on Carbohydrates. His wife is an employee of Unilever Canada.

## References

[1] Bray GA. Fructose: pure, white, and deadly? Fructose, by any other name, is a health hazard. J Diabetes Sci Technol (2010) 4:1003–7. 10.1177/193229681000400432 20663467 PMC2909535

[2] Lustig RH. Fructose: it’s “alcohol without the buzz”. Adv Nutr (2013) 4:226–35. 10.3945/an.112.002998 23493539 PMC3649103

[3] DiNicolantonio JJ O’Keefe JH Lucan SC. Added fructose: a principal driver of type 2 diabetes mellitus and its consequences. Mayo Clin Proc (2015) 90(3):372–81. 10.1016/j.mayocp.2014.12.019 25639270

[4] Bray GA Nielsen SJ Popkin BM. Consumption of high-fructose corn syrup in beverages may play a role in the epidemic of obesity. Am J Clin Nutr (2004) 79:537–43. 15051594 10.1093/ajcn/79.4.537

[5] Lee WN Bassilian S Ajie HO Schoeller DA Edmond J Bergner EA In vivo measurement of fatty acids and cholesterol synthesis using D2O and mass isotopomer analysis. Am J Physiol (1994) 266:E699–708. 8203508 10.1152/ajpendo.1994.266.5.E699

[6] Brunengraber DZ Mccabe BJ Kasumov T Alexander JC Chandramouli V Previs SF. Influence of diet on the modeling of adipose tissue triglycerides during growth. Am J Physiol Endocrinol Metab (2003) 285:E917–25. 10.1152/ajpendo.00128.2003 12799315

[7] Dolan LC Potter SM Burdock GA. Evidence-based review on the effect of normal dietary consumption of fructose on blood lipids and body weight of overweight and obese individuals. Crit Rev Food Sci Nutr (2010) 50:889–918. 10.1080/10408398.2010.512990 21108071

[8] Cozma AI Sievenpiper JL De Souza RJ Chiavaroli L Ha V Wang DD Effect of fructose on glycemic control in diabetes: a systematic review and meta-analysis of controlled feeding trials. Diabetes Care (2012) 35:1611–20 10.2337/dc12-0073 22723585 PMC3379616

[9] Ha V Sievenpiper JL De Souza RJ Chiavaroli L Wang DD Cozma AI Effect of fructose on blood pressure: a systematic review and meta-analysis of controlled feeding trials. Hypertension (2012) 59:787–95. 10.1161/HYPERTENSIONAHA.111.182311 22331380

[10] Sievenpiper JL De Souza RJ Mirrahimi A Yu ME Carleton AJ Beyene J Effect of fructose on body weight in controlled feeding trials: a systematic review and meta-analysis. Ann Intern Med (2012) 156:291–304. 10.7326/0003-4819-156-4-201202210-00007 22351714

[11] Wang DD Sievenpiper JL De Souza RJ Chiavaroli L Ha V Cozma AI The effects of fructose intake on serum uric acid vary among controlled dietary trials. J Nutr (2012) 142:916–23. 10.3945/jn.111.151951 22457397 PMC3327749

[12] Chiu S Sievenpiper JL De Souza RJ Cozma AI Mirrahimi A Carleton AJ Effect of fructose on markers of non-alcoholic fatty liver disease (NAFLD): a systematic review and meta-analysis of controlled feeding trials. Eur J Clin Nutr (2014) 68:416–23. 10.1038/ejcn.2014.8 24569542 PMC3975811

[13] David Wang D Sievenpiper JL De Souza RJ Cozma AI Chiavaroli L Ha V Effect of fructose on postprandial triglycerides: a systematic review and meta-analysis of controlled feeding trials. Atherosclerosis (2014) 232:125–33. 10.1016/j.atherosclerosis.2013.10.019 24401226

[14] Marriott BP Cole N Lee E. National estimates of dietary fructose intake increased from 1977 to 2004 in the United States. J Nutr (2009) 139:1228S–35S. 10.3945/jn.108.098277 19403716

[15] Kaufmann NA Poznanski R Blondheim SH Stein Y Effect of fructose, glucose, sucrose and starch on serum lipids in carbohydrate induced hypertriglyceridemia and in normal subjects. Isr J Med Sci (1966) 2:715–26. 5956687

[16] Nestel PJ Carroll KF Havenstein N Plasma triglyceride response to carbohydrates, fats and caloric intake. Metabolism (1970) 19:1–18 10.1016/0026-0495(70)90112-5 5410659

[17] Akerblom HK Siltanen I Kallio AK Does dietary fructose affect the control of diabetes in children? Acta Med Scand Suppl (1972) 542:195–202. 4516490 10.1111/j.0954-6820.1972.tb05335.x

[18] Nikkila EA Kekki M Effects of dietary fructose and sucrose on plasma triglyceride metabolism in patients with endogenous hypertriglyceridemia. Acta Med Scand Suppl (1972) 542:221–7. 4516491 10.1111/j.0954-6820.1972.tb05338.x

[19] Pelkonen R Aro A Nikkila EA Metabolic effects of dietary fructose in insulin dependent diabetes of adults. Acta Med Scand Suppl (1972) 542: 187–93. 4579750 10.1111/j.0954-6820.1972.tb05334.x

[20] Forster H Heller G [Studies on the significance of carbohydrates in a fully synthetic fat-free diet]. Dtsch Med Wochenschr (1973) 98:1156–63 10.1055/s-0028-1106986 4711888

[21] Huttunen JK Makinen KK Scheinin A. Turku sugar studies XI. Effects of sucrose, fructose and xylitol diets on glucose, lipid and urate metabolism. Acta Odontol Scand (1976) 34:345–51. 10.3109/00016357609004646 1070904

[22] Turner JL Bierman EL Brunzell JD Chait A. Effect of dietary fructose on triglyceride transport and glucoregulatory hormones in hypertriglyceridemic men. Am J Clin Nutr (1979) 32:1043–50. 433820 10.1093/ajcn/32.5.1043

[23] Beck-Nielsen H Pedersen O Lindskov HO. Impaired cellular insulin binding and insulin sensitivity induced by high-fructose feeding in normal subjects. Am J Clin Nutr (1980) 33:273–8. 6986758 10.1093/ajcn/33.2.273

[24] Cybulska B Naruszewicz M. The effect of short-term and prolonged fructose intake on VLDL-TG and relative properties on apo CIII1 and apo CII in the VLDL fraction in type IV hyperlipoproteinaemia. Nahrung (1982) 26:253–61. 10.1002/food.19820260306 7110307

[25] Hallfrisch J Ellwood KC Michaelis OET Reiser S O’Dorisio TM Prather ES. Effects of dietary fructose on plasma glucose and hormone responses in normal and hyperinsulinemic men. J Nutr (1983) 113:1819–26. 6350544 10.1093/jn/113.9.1819

[26] Bossetti BM Kocher LM Moranz JF Falko JM. The effects of physiologic amounts of simple sugars on lipoprotein, glucose, and insulin levels in normal subjects. Diabetes Care (1984) 7:309–12. 10.2337/diacare.7.4.309 6381003

[27] Crapo PA Kolterman OG. The metabolic effects of 2-week fructose feeding in normal subjects. Am J Clin Nutr (1984) 39:525–34. 6369956 10.1093/ajcn/39.4.525

[28] Bantle JP Laine DC Thomas JW. Metabolic effects of dietary fructose and sucrose in types I and II diabetic subjects. JAMA (1986) 256:3241–6. 10.1001/jama.1986.03380230065027 3783868

[29] Crapo PA Kolterman OG Henry RR. Metabolic consequence of two-week fructose feeding in diabetic subjects. Diabetes Care (1986) 9:111–9. 10.2337/diacare.9.2.111 3516605

[30] Rizkalla SW Baigts F Fumeron F Rabillon B Bayn P Ktorza A Comparative effects of several simple carbohydrates on erythrocyte insulin receptors in obese subjects. Pharmacol Biochem Behav (1986) 25:681–8. 10.1016/0091-3057(86)90159-0 3534894

[31] McAteer EJ O’Reilly G Hadden DR. The effects of one month high fructose intake on plasma glucose and lipid levels in non-insulin-dependent diabetes. Diabet Med (1987) 4:62–4. 10.1111/j.1464-5491.1987.tb00831.x 2951223

[32] Osei K Falko J Bossetti BM Holland GC. Metabolic effects of fructose as a natural sweetener in the physiologic meals of ambulatory obese patients with type II diabetes. Am J Med (1987) 83:249–55. 10.1016/0002-9343(87)90693-0 3618627

[33] Grigoresco C Rizkalla SW Halfon P Bornet F Fontvieille AM Bros M Lack of detectable deleterious effects on metabolic control of daily fructose ingestion for 2 mo in NIDDM patients. Diabetes Care (1988) 11:546–50. 10.2337/diacare.11.7.546 3203571

[34] Koh ET Ard NF Mendoza F. Effects of fructose feeding on blood parameters and blood pressure in impaired glucose-tolerant subjects. J Am Diet Assoc (1988) 88:932–8. 3294273

[35] Anderson JW Story LJ Zettwoch NC Gustafson NJ Jefferson BS. Metabolic effects of fructose supplementation in diabetic individuals. Diabetes Care (1989) 12:337–44. 10.2337/diacare.12.5.337 2721342

[36] Osei K Bossetti B. Dietary fructose as a natural sweetener in poorly controlled type 2 diabetes: a 12-month crossover study of effects on glucose, lipoprotein and apolipoprotein metabolism. Diabet Med (1989) 6:506–11. 10.1111/j.1464-5491.1989.tb01218.x 2527132

[37] Reiser S Powell AS Scholfield DJ Panda P Ellwood KC Canary JJ. Blood lipids, lipoproteins, apoproteins, and uric acid in men fed diets containing fructose or high-amylose cornstarch. Am J Clin Nutr (1989) 49:832–9. 2497634 10.1093/ajcn/49.5.832

[38] Thorburn AW Crapo PA Beltz WF Wallace P Witztum JL Henry RR. Lipid metabolism in non-insulin-dependent diabetes: effects of long-term treatment with fructose-supplemented mixed meals. Am J Clin Nutr (1989) 50:1015–22. 2683716 10.1093/ajcn/50.5.1015

[39] Bantle JP Swanson JE Thomas W Laine DC. Metabolic effects of dietary fructose in diabetic subjects. Diabetes Care (1992) 15:1468–76. 10.2337/diacare.15.11.1468 1468273

[40] Swanson JE Laine DC Thomas W Bantle JP Metabolic effects of dietary fructose in healthy subjects. Am J Clin Nutr (1992) 55:851–6. 1550068 10.1093/ajcn/55.4.851

[41] Koivisto VA Yki-Jarvinen H. Fructose and insulin sensitivity in patients with type 2 diabetes. J Intern Med (1993) 233:145–53. 10.1111/j.1365-2796.1993.tb00667.x 8433075

[42] Malerbi DA Paiva ES Duarte AL Wajchenberg BL. Metabolic effects of dietary sucrose and fructose in type II diabetic subjects. Diabetes Care (1996) 19:1249–56. 10.2337/diacare.19.11.1249 8908389

[43] Bantle JP Raatz SK Thomas W Georgopoulos A. Effects of dietary fructose on plasma lipids in healthy subjects. Am J Clin Nutr (2000) 72:1128–34. 11063439 10.1093/ajcn/72.5.1128

[44] Sunehag AL Toffolo G Treuth MS Butte NF Cobelli C Bier DM Effects of dietary macronutrient content on glucose metabolism in children. J Clin Endocrinol Metab (2002) 87:5168–78. 10.1210/jc.2002-020674 12414888

[45] Treuth MS Sunehag AL Trautwein LM Bier DM Haymond MW Butte NF. Metabolic adaptation to high-fat and high-carbohydrate diets in children and adolescents. Am J Clin Nutr (2003) 77:479–89. 12540411 10.1093/ajcn/77.2.479

[46] Vaisman N Niv E Izkhakov Y. Catalytic amounts of fructose may improve glucose tolerance in subjects with uncontrolled non-insulin-dependent diabetes. Clin Nutr (2006) 25:617–21. 10.1016/j.clnu.2005.11.013 16403592

[47] Sunehag AL Toffolo G Campioni M Bier DM Haymond MW. Short-term high dietary fructose intake had no effects on insulin sensitivity and secretion or glucose and lipid metabolism in healthy, obese adolescents. J Pediatr Endocrinol Metab (2008) 21:225–35. 18540249 10.1515/jpem.2008.21.3.225

[48] Swarbrick MM Stanhope KL Elliott SS Graham JL Krauss RM Christiansen MP Consumption of fructose-sweetened beverages for 10 weeks increases postprandial triacylglycerol and apolipoprotein-B concentrations in overweight and obese women. Br J Nutr (2008) 100:947–52. 10.1017/S0007114508968252 18384705 PMC3038917

[49] Stanhope KL Schwarz JM Keim NL Griffen SC Bremer AA Graham JL Consuming fructose-sweetened, not glucose-sweetened, beverages increases visceral adiposity and lipids and decreases insulin sensitivity in overweight/obese humans. J Clin Invest (2009) 119:1322–34 10.1172/JCI37385 19381015 PMC2673878

[50] Ngo Sock ET Le KA Ith M Kreis R Boesch C Tappy L. Effects of a short-term overfeeding with fructose or glucose in healthy young males. Br J Nutr (2010) 103:939–43. 10.1017/S0007114509992819 19930762

[51] Aeberli I Gerber PA Hochuli M Kohler S Haile SR Gouni-Berthold I Low to moderate sugar-sweetened beverage consumption impairs glucose and lipid metabolism and promotes inflammation in healthy young men: a randomized controlled trial. Am J Clin Nutr (2011) 94:479–85. 10.3945/ajcn.111.013540 21677052

[52] Madero M Arriaga JC Jalal D Rivard C Mcfann K Perez-Mendez O The effect of two energy-restricted diets, a low-fructose diet versus a moderate natural fructose diet, on weight loss and metabolic syndrome parameters: a randomized controlled trial. Metabolism (2011) 60:1551–9. 10.1016/j.metabol.2011.04.001 21621801

[53] Silbernagel G Machann J Unmuth S Schick F Stefan N Haring HU Effects of 4-week very-high-fructose/glucose diets on insulin sensitivity, visceral fat and intrahepatic lipids: an exploratory trial. Br J Nutr (2011) 106:79–86. 10.1017/S000711451000574X 21396140

[54] Stanhope KL Bremer AA Medici V Nakajima K Ito Y Nakano T Consumption of fructose and high fructose corn syrup increase postprandial triglycerides, LDL-cholesterol, and apolipoprotein-B in young men and women. J Clin Endocrinol Metab (2011) 96:E1596–605. 10.1210/jc.2011-1251 21849529 PMC3200248

[55] Brymora A Flisinski M Johnson RJ Goszka G Stefanska A Manitius J. Low-fructose diet lowers blood pressure and inflammation in patients with chronic kidney disease. Nephrol Dial Transplant (2012) 27:608–12. 10.1093/ndt/gfr223 21613382 PMC3350341

[56] Cox CL Stanhope KL Schwarz JM Graham JL Hatcher B Griffen SC Consumption of fructose- but not glucose-sweetened beverages for 10 weeks increases circulating concentrations of uric acid, retinol binding protein-4, and gamma-glutamyl transferase activity in overweight/obese humans. Nutr Metab (Lond) (2012) 9:68 10.1186/1743-7075-9-68 22828276 PMC3463498

[57] Aeberli I Hochuli M Gerber PA Sze L Murer SB Tappy L Moderate amounts of fructose consumption impair insulin sensitivity in healthy young men: a randomized controlled trial. Diabetes Care (2013) 36:150–6 10.2337/dc12-0540 22933433 PMC3526231

[58] Johnston RD Stephenson MC Crossland H Cordon SM Palcidi E Cox EF No difference between high-fructose and high-glucose diets on liver triacylglycerol or biochemistry in healthy overweight men. Gastroenterology (2013) 145(1016–1025):e1012. 10.1053/j.gastro.2013.07.012 23872500

[59] Lecoultre V Egli L Carrel G Theytaz F Kreis R Schneiter P Effects of fructose and glucose overfeeding on hepatic insulin sensitivity and intrahepatic lipids in healthy humans. Obesity (Silver Spring) (2013) 21:782–5. 10.1002/oby.20377 23512506

[60] Tobias LE A Comparison of the Effect of Consuming a Fructose-, Glucose-, or Aspartame-Sweetened Beverage on Ad libitum Caloric Intake [Doctoral Dissertation]. Seattle (WA): University of Washington (2013).

[61] Heden TD Liu Y Park YM Nyhoff LM Winn NC Kanaley JA. Moderate amounts of fructose- or glucose-sweetened beverages do not differentially alter metabolic health in male and female adolescents. Am J Clin Nutr (2014) 100:796–805. 10.3945/ajcn.113.081232 25030782 PMC4135490

[62] Jin R Welsh JA Le NA Holzberg J Sharma P Martin DR Dietary fructose reduction improves markers of cardiovascular disease risk in Hispanic-American adolescents with NAFLD. Nutrients (2014) 6:3187–201. 10.3390/nu6083187 25111123 PMC4145302

[63] Mark AB Poulsen MW Andersen S Andersen JM Bak MJ Ritz C Consumption of a diet low in advanced glycation end products for 4 weeks improves insulin sensitivity in overweight women. Diabetes Care (2014) 37:88–95. 10.2337/dc13-0842 23959566

[64] Le KA Faeh D Stettler R Ith M Kreis R Vermathen P A 4-wk high-fructose diet alters lipid metabolism without affecting insulin sensitivity or ectopic lipids in healthy humans. Am J Clin Nutr (2006) 84:1374–9. 17158419 10.1093/ajcn/84.6.1374

[65] Le KA Ith M Kreis R Faeh D Bortolotti M Tran C Fructose overconsumption causes dyslipidemia and ectopic lipid deposition in healthy subjects with and without a family history of type 2 diabetes. Am J Clin Nutr (2009) 89:1760–5. 10.3945/ajcn.2008.27336 19403641

[66] Sobrecases H Le KA Bortolotti M Schneiter P Ith M Kreis R Effects of short-term overfeeding with fructose, fat and fructose plus fat on plasma and hepatic lipids in healthy men. Diabetes Metab (2010) 36:244–6. 10.1016/j.diabet.2010.03.003 20483648

[67] Blayo A Fontveille AM Rizkalla S Bruzzo F Slama G Effets metaboliques de la consommation quotidienne pendant un an de saccharose ou de fructose par des diabetiques. Medecine et Nutrition (1990) 26:909–13.

[68] Thorburn AW Crapo PA Griver K Wallace P Henry RR Long-term effects of dietary fructose on carbohydrate metabolism in non-insulin-dependent diabetes mellitus. Metabolism (1990) 39(1):58–63 10.1016/0026-0495(90)90148-6 2403621

[69] Crapo PA Reaven G Olefsky J. Plasma glucose and insulin responses to orally administered simple and complex carbohydrates. Diabetes (1976) 25:741–7. 10.2337/diab.25.9.741 955301

[70] Jenkins DJ Wolever TM Taylor RH Barker H Fielden H Baldwin JM Glycemic index of foods: a physiological basis for carbohydrate exchange. Am J Clin Nutr (1981) 34:362–6. 6259925 10.1093/ajcn/34.3.362

[71] Atkinson FS Foster-Powell K Brand-Miller JC. International tables of glycemic index and glycemic load values: 2008. Diabetes Care (2008) 31:2281–3. 10.2337/dc08-1239 18835944 PMC2584181

[72] Archer E Hand GA Blair SN. Validity of U.S. nutritional surveillance: National Health and Nutrition Examination Survey caloric energy intake data, 1971-2010. PLoS One (2013) 8:e76632. 10.1371/journal.pone.0076632 24130784 PMC3793920

[73] Fereday N Forber G Firardello S Midgley C Nutt T Powell N HFCS Industry Annual Review – A Year of Changing Expectations. Oxford: L. International (2007).

[74] Sievenpiper JL Carleton AJ Chatha S Jiang HY De Souza RJ Beyene J Heterogeneous effects of fructose on blood lipids in individuals with type 2 diabetes: systematic review and meta-analysis of experimental trials in humans. Diabetes Care (2009) 32:1930–7. 10.2337/dc09-0619 19592634 PMC2752906

[75] Sievenpiper JL Chiavaroli L De Souza RJ Mirrahimi A Cozma AI Ha V ‘Catalytic’ doses of fructose may benefit glycaemic control without harming cardiometabolic risk factors: a small meta-analysis of randomised controlled feeding trials. Br J Nutr (2012) 108:418–23 10.1017/S000711451200013X 22354959 PMC3411192

[76] Livesey G Taylor R. Fructose consumption and consequences for glycation, plasma triacylglycerol, and body weight: meta-analyses and meta-regression models of intervention studies. Am J Clin Nutr (2008) 88:1419–37. 18996880 10.3945/ajcn.2007.25700

[77] Sievenpiper JL Toronto DKS Clinical Trials U Fructose: where does the truth lie? J Am Coll Nutr (2012) 31:149–51 10.1080/07315724.2012.10720021 23204150

